# Impact of a family support intervention on hospitalization costs and hospital readmissions among ICU patients at high risk of death or severe functional impairment

**DOI:** 10.1186/s13613-024-01344-9

**Published:** 2024-07-02

**Authors:** Sarah K. Andersen, Chung-Chou H. Chang, Robert M. Arnold, Caroline Pidro, Joseph M. Darby, Derek C. Angus, Douglas B. White, the Pairing Re-engineered Intensive Care Teams with Nurse-driven Emotional Support, Relationship building (PARTNER) Investigators

**Affiliations:** 1 Program on Ethics and Decision Making, Department of Critical Care Medicine, The Clinical Research, Investigation, and Systems Modeling of Acute Illness (CRISMA) Center, University of Pittsburgh School of Medicine, 3550 Terrace St, Scaife Hall, Room 608, Pittsburgh, PA 15261 USA; 2 Department of Critical Care Medicine, Faculty of Medicine and Dentistry, University of Alberta, Edmonton, AB Canada; 3 Department of Critical Care Medicine, The CRISMA Center, University of Pittsburgh School of Medicine, Pittsburgh, PA USA; 4 Department of Medicine, University of Pittsburgh School of Medicine, Pittsburgh, PA USA; 5 Section of Palliative Care and Medical Ethics, Department of General Internal Medicine, University of Pittsburgh School of Medicine, Pittsburgh, PA USA; 6 Palliative and Supportive Institute, UPMC Health System, Pittsburgh, PA USA; 7 Department of Critical Care Medicine, University of Pittsburgh School of Medicine, Pittsburgh, PA USA

**Keywords:** Critical care, Communication, Cost-effectiveness, Patient centered care, Health services research

## Abstract

**Background:**

Patients with advanced critical illness often receive more intensive treatment than they would choose for themselves, which contributes to high health care costs near the end of life. The purpose of this study was to determine whether a family support intervention delivered by the interprofessional ICU team decreases hospitalization costs and hospital readmissions among critically ill patients at high risk of death or severe functional impairment.

**Results:**

We examined index hospitalization costs as well as post-discharge utilization of acute care hospitals, rehabilitation and skilled nursing facilities, and hospice services for the PARTNER trial, a multicenter, stepped-wedge, cluster randomized trial of an interprofessional ICU family support intervention. We determined patients’ total controllable and direct variable costs using a computerized accounting system. We determined post-discharge resource utilization (as defined above) by structured telephone interview at 6-month follow-up. We used multiple variable regression modelling to compare outcomes between groups. Compared to usual care, the PARTNER intervention resulted in significantly lower total controllable costs (geometric mean: $26,529 vs $32,105; log-linear coefficient: − 0.30; 95% CI − 0.49, − 0.11) and direct variable costs ($3912 vs $6034; − 0.33; 95% CI − 0.56, − 0.10). A larger cost reduction occurred for decedents ($20,304 vs. $26,610; − 0.66; 95% CI − 1.01, − 0.31) compared to survivors ($31,353 vs. $35,015; − 0.15; 95% CI − 0.35,0.05). A lower proportion in the intervention arm were re-admitted to an acute care hospital (34.9% vs 45.1%; 0.66; 95% CI 0.56, 0.77) or skilled nursing facility (25.3% vs 31.6%; 0.63; 95% CI 0.47, 0.84).

**Conclusions:**

A family support intervention delivered by the interprofessional ICU team significantly decreased index hospitalization costs and readmission rates over 6-month follow-up.

*Trial registration* Trial registration number: NCT01844492

**Supplementary Information:**

The online version contains supplementary material available at 10.1186/s13613-024-01344-9.

## Introduction

Approximately 500,000 Americans die annually following admission to an intensive care unit (ICU) [1]. Because these patients typically lack decision-making capacity due to advanced illness or neuroactive medications, clinicians turn to patients’ surrogate decision-makers to assist with decisions regarding goals of care [2–4]. However, communication breakdowns are well documented [5–15] and surrogates report that the experience of authorizing withdrawal of life support is psychologically and emotionally difficult [16–19]. Consequently, patients near the end of life often receive more invasive, burdensome treatments than they would choose for themselves [20–23]. These problems threaten the ethical goal of patient-centered care and also contribute to the high costs of end-of-life care in the United States [24, 25]

We previously reported the results of a multicenter trial of a family support intervention delivered by the interprofessional ICU team [26]. The intervention improved surrogates’ ratings of the patient- and family-centeredness of care, decreased ICU and hospital length of stay, and did not impact surrogates’ psychological symptom burden at 6-month follow-up. The intervention was designed to be low-cost to deliver, to increase the role of nurses in supporting families, and to be feasible to deploy in most U.S. hospitals.

In our initial publication of the PARTNER trial, we did not report the effects of the intervention on costs of the index hospitalization or on hospital readmissions. Understanding whether the observed improvements in patient- and family-centeredness of care were accompanied by cost savings (or cost increases) is important for health systems and payers who may consider adopting the PARTNER intervention. Few studies of family support interventions have conducted a costing assessment, which we believe to be an important piece of establishing real-world benefit. In general, an intervention that simultaneously improves outcomes and decreases costs has high potential for broad adoption. If an intervention improves outcomes but increases costs, a detailed understanding of the magnitude of costs compared to benefits is often needed to inform funding decisions. We therefore determined the impact of the PARTNER intervention on costs during patients’ index hospitalization and on survivors’ utilization of acute care hospitals, skilled nursing and rehabilitation facilities, and hospice through 6-month follow-up.

## Methods

### Study design

From July 2012 to February 2016, we conducted a multicenter, stepped-wedge cluster randomized trial comparing a multicomponent family support intervention delivered by the interprofessional ICU team called PARTNER **(PA**iring **R**e-engineered ICU **T**eams with **N**urse-driven **E**motional Support and **R**elationship-building) to usual care. The study protocol and results of the primary analyses have been previously published [26]. The intervention was determined to be quality improvement because it was designed to improve the implementation of care practices recommended in professional society practice statements [27–29]. Surrogates provided informed consent for participation in long-term follow-up and were informed of the quality improvement project. The study was reviewed and approved by the University of Pittsburgh Institutional Review Board (PRO13020304), the UPMC Quality Improvement Committee, and the leadership of participating ICUs.

### Setting and eligibility criteria

Patients were recruited from five ICUs from five hospitals within the University of Pittsburgh Medical Center (UPMC) Health System: a neurological ICU, a surgical transplant ICU, two medical surgical ICUs, and a medical ICU. Details about physician and nurse staffing models were previously described.

Patient inclusion criteria included: (1) age greater than or equal to 18 years; (2) lack of decision-making capacity based on the clinical examination of the patient’s attending physician; and (3) at least one of the following clinical characteristics: (a) receipt of mechanical ventilation for ≥ 4 consecutive days; (b) an estimated ≥ 40% chance of hospital mortality as judged by the patient’s attending physician; or (c) an estimated ≥ 40% chance of severe long term functional impairment as judged by the patient’s attending physician. Patients were excluded if they lacked a surrogate decision-maker or were receiving only comfort-focused treatment at the time of screening. We enrolled one surrogate decision-maker per patient, based on the family’s judgement of who was acting as the main surrogate for the patient.

### Randomization and study intervention

We used a computer-generated randomization scheme to determine the order in which sites transitioned from control phase (usual care) to intervention phase, in 6-month intervals. A detailed description of the PARTNER intervention has been previously published [26]. Briefly, the intervention is grounded in the Cognitive-Emotional Decision Making framework [30]. This framework conceptualizes the challenges of medical decision making as not only cognitive in nature, but also related to the affective and psychological difficulty of making high-stakes health decisions for a critically ill loved one [31]. The PARTNER intervention therefore was designed to attend to the cognitive, affective, and psychological challenges that surrogates face, through delivering guideline-recommended emotional support and ensuring frequent clinician-family communication [27–29]. The intervention was deployed by members of the existing ICU interprofessional team; it was overseen by 4–6 nurses in each ICU, called the PARTNER nurses, who were nominated by their ICU director and who received additional training, as described below.

There were three main components to the PARTNER intervention. First, the PARTNER nurses in each ICU received advanced communication skills training to provide intensive support to surrogates. This training was delivered through a 12-h course that employed a series didactic teaching, modelling of the communication skills by an expert instructor, extensive skills practice with trained medical actors, and personalized feedback provided by the instructor. Second, each ICU revised their care processes to deploy a structured family support pathway in which the PARTNER nurses met with families daily according to a standardized protocol and arranged interdisciplinary clinician-family meetings within 48 h of enrollment and every 5–7 days thereafter. Third, a quality improvement specialist from the UPMC health system provided each ICU with intensive implementation support to assist them in adopting the family support pathway in their ICU.

### Data collection

We used the UPMC health system electronic health record to obtain the following information: patient demographics, primary diagnosis, admission source, severity of illness on ICU admission as determined by a modified Simplified Acute Physiology Score (SAPS) III [32], comorbidities measured with the Elixhauser score [33], and disposition upon hospital discharge. We determined hospitalization costs using the UPMC health system computerized cost accounting, described below. Research staff masked to treatment group conducted telephone interviews with surrogate decision-makers 6 months after the patient’s hospital discharge, during which they ascertained surrogates’ demographic information and information about patients’ post-discharge use of acute inpatient care, skilled nursing and rehabilitation facilities, and hospice services.

### Outcomes

We quantified hospitalization costs by determining both the total controllable hospitalization costs and the direct variable costs of each admission. To calculate total controllable hospitalization costs, the UPMC computerized cost accounting system assigns specific costs to each service based on hospital expenses. UPMC developed this activity-based costing (ABC) system to align costs with patients based on actual utilization of resources. Direct expenses, such as blood products, drugs, and supplies, are allocated to individual patients based on usage. Departmental labor, including clinician services, and other fixed clinically oriented expenses such as utilities and facility maintenance are calculated as global costs and allocated to patients using specific cost drivers, such minutes on a nursing unit or time in an OR. This costing method excludes fully indirect expenses such as organizational business and administrative activities (i.e., marketing, legal, finance, human resources, talent acquisition) and information technology support. Because this costing method excludes these categories of fixed costs, which account for approximately 25% of total hospitalization costs, the label “total controllable hospitalization costs” is more accurate than total hospitalization costs.

To calculate direct-variable costs, we removed the fixed costs of overhead that are not related to patient throughput, determined through individual departmental usage patterns [34] and aggregated each patient’s total service specific costs. The categories included in direct variable costs consist of blood products (the cost of all blood products and related services), drugs (the costs of all drugs directly attributable to that encounter), and supplies (the costs of all supplies directly attributable to that patient). Further details of the activity-based costing system can be found at: https://www.healthcatalyst.com/success_stories/activity-based-costing-in-healthcare-service-lines-upmc.

To determine patients’ post-discharge inpatient and hospice care utilization, trained research staff used an established interview guide, which they administered to surrogates by telephone 6 months after hospital discharge. Interviewers determined whether the patient was subsequently admitted to an acute care hospital, a long-term acute care hospital, a skilled nursing facility, or a rehabilitation facility. Interviewers also determined whether patients utilized hospice services during the 6-month follow-up period. Emergency department visits, outpatient visits, and non-hospice homecare use were excluded.

As previously reported [26], we calculated the cost per patient to deploy the intervention by summing the overall costs to deploy the intervention and dividing this by the number of patients in the intervention arm. Cost data was analyzed according to 2012–2016 prices, unadjusted for inflation. Time was included in the multiple variable model to account for the potential for confounding due to cost increases over the study period.

### Statistical analysis

All analyses were performed on an intention-to-treat basis using Stata 15 software [35]. The individual ICU was the unit of randomization and the individual patient was the unit of analysis. Because of the nonnegative and skewed nature of cost data, we used log-linear mixed modeling with log-transformed costs to examine the intervention’s impact on total controllable and direct-variable hospitalization costs. We incorporated an ICU-level random effect to address clustering by ICU and random slopes of time to account for temporal effects, as delineated by Hussey and Hughes [36, 37]. We prespecified that all analyses would be adjusted for patient age, sex, race, modified Simplified Acute Physiology Score (SAPS) III, Elixhauser index, mechanical ventilation usage, admission source, and primary diagnosis. We also prespecified that we would conduct stratified analyses by whether the patient survived to hospital discharge.

To determine whether the intervention affected utilization of acute care, skilled nursing, and rehabilitation facilities, and hospice after the index hospitalization, we used multivariable logistic regression with robust standard errors for site clustering. We selected this modelling approach rather than mixed effects modelling because the time effect was not statistically significant and because the site effect led to model instability. We adjusted for the same covariates noted above. We assessed the stability of final models using routine model diagnostics to identify potential outliers and/or influential observations.

## Results

As previously described, 1420 patients met entry criteria and were included in the trial. Table 1 shows the demographic characteristics of the patients. There were baseline differences between treatment groups, including higher age, acute severity of illness, and number of chronic comorbidities in the intervention arm. Surrogates for 1106 patients agreed to be contacted for long-term follow-up and 809 of these (73%) completed long-term follow up; Table 2 summarizes their demographic characteristics. The overall 6-month mortality rate among patients in the trial was 56.7% (805/1420).

**Table 1:** Characteristics of enrolled patients

Patient characteristic	Control (n = 873)	Intervention (n = 547)	p-value^a^
Age			
Mean (SD)	63.3 (15.5)	67.5 (14.9)	< 0.001
Median (IQR)	65 (20)	68 (21)	
Female, count (%)	405 (46.4)	290 (53.0)	0.02
Race, count (%)			
White	708 (81.1)	459 (83.9)	0.30
Black	64 (7.3)	43 (7.9)	
Hispanic	2 (0.2)	0	
Other	9 (1.0)	4 (0.7)	
Not documented	90 (10.3)	41 (7.5)	
Primary diagnosis, count (%)			
Cardiovascular	36 (4.1)	33 (6.0)	< 0.01
Pulmonary	138 (15.9)	107 (19.6)	
GI	94 (10.8)	49 (9.0)	
Toxicology	38 (4.4)	18 (3.3)	
Infection/sepsis	212 (24.4)	159 (29.1)	
Neurological	173 (19.9)	106 (19.4)	
Oncological	59 (6.8)	18 (3.3)	
Other	118 (13.6)	56 (10.3)	
Admission source, count (%)			
Direct	224 (25.7)	50 (9.1)	< 0.001
Emergency	549 (62.9)	422 (77.2)	
Other hospital	100 (11.5)	73 (13.4)	
Skilled nursing facility	0	2 (0.4)	
Modified SAPS^b^ III score, mean (SD)	49.4 (12.0)	51.0 (11.8)	0.02
Elixhauser comorbidity index (count; range 0–29), mean (SD)	5.1 (2.5)	5.8 (2.4)	< 0.001
On mechanical ventilation during hospitalization, count (%)	759 (86.9)	479 (87.6)	0.73

^a^From Student’s t-test or Pearson’s chi-squared test; ^b^*SAPS* = simplified acute physiology score

**Table 2:** Characteristics of enrolled surrogates

Surrogate characteristic	Control (n = 677)	Intervention (n = 429)	p-value^a^
Age, mean (SD)	56.4 (13.6)	57.1 (13.7)	0.46
Female, count (%)	480 (70.9)	284 (66.2)	0.06
Race, count (%)			
White	559 (82.6)	383 (89.3)	0.17
Black	37 (5.5)	36 (8.4)	
Hispanic	5 (0.7)	0 (0)	
Asian	6 (0.9)	2 (0.5)	
Multiethnic	3 (0.4)	2 (0.5)	
Not documented	67 (9.9)	6 (1.4)	
Relationship to patient			
Spouse/partner	295 (43.6)	161 (37.5)	0.04
Parent	63 (9.3)	28 (6.5)	
Child	197 (29.1)	163 (38.0)	
Sibling	81 (12.0)	53 (12.4)	
Other relative	18 (2.7)	12 (2.8)	
Other relationship	20 (3.0)	9 (2.1)	
Not documented	3 (0.4)	3 (0.7)	

^a^From Student’s t-test or Pearson’s chi-squared test

Table 3 summarizes the main cost analyses of the trial. Because the UPMC activity-based costing system was put into place after the trial commenced, cost data were available for 1012 of 1420 patients (71.3%; Supplemental Table 1). The intervention resulted in significantly lower total controllable hospitalization costs ($26,529 vs $32,105; unadjusted p-value 0.005; adjusted log-linear model coefficient: − 0.30; 95% CI − 0.49 to − 0.11; p = 0.002). A larger reduction in total controllable hospitalization costs occurred among patients who died during index hospitalization ($20,304 vs. $26,610; unadjusted p-value 0.028; log-linear model coefficient: − 0.66; 95% CI − 1.01 to − 0.31; p < 0.001) compared to those who survived to hospital discharge ($31,353 vs. $35,015; unadjusted p-value 0.173; adjusted log-linear model coefficient: − 0.15; 95% CI − 0.35 to 0.05; p = 0.135). The intervention also significantly decreased direct variable costs, both for decedents and survivors, as summarized in Table 3.

**Table 3:** Costs of index hospitalization (N = 1012)

Healthcare utilization	Control (n = 484) US$, geometric mean	Intervention (n = 528) US$, geometric mean	Unadjusted p-values^a^	Intervention effect (95% CI)^b^	Adjusted p-value^b^
Total controllable hospitalization cost	32,104.63	26,529.28	0.005	β − 0.30^c^ (− 0.49 to − 0.11)	0.002
Decedents	26,610.10	20,303.79	0.0279	β − 0.66^c^ (− 1.01 to − 0.31)	< 0.001
Survivors	35,014.63	31,352.60	0.1725	β − 0.15^c^ (− 0.35 to 0.05)	0.135
Direct variable cost^d^	6034.37	3912.25	< 0.001	β − 0.33^c^ (− 0.56 to − 0.10)	0.005
Decedents	5570.70	3611.21	0.0038	β − 0.61^c^ (− 1.03 to − 0.20)	0.004
Survivors	6270.83	4112.51	0.0001	β − 0.27^c^ (− 0.52 to − 0.02)	0.035

^a^From Student’s t-test for log-transformed geometric mean costs

^b^All models were adjusted for patient’s age, sex, race, modified SAPS III, Elixhauser index, mechanical ventilation usage, primary diagnosis, admission source, site and time of enrollment

^c^Results from log-linear regression modeling. Outcome was log-transformed

^d^Exclude patients with $0 direct cost (n = 24; 22 Control and 2 Intervention)

Table 4 summarizes the post-discharge utilization of inpatient and hospice services for patients who survived the index hospitalization, stratified by treatment group. Among the 809 patients whose surrogate completed the 6-month follow-up call (Supplemental Table 2), 241 died during the index hospitalization and therefore had no post-discharge health care utilization. Among the 568 survivors of the index hospitalization, a lower proportion of patients in the intervention arm were subsequently re-admitted to an acute care hospital (34.9% vs 45.1%; adjusted OR: 0.66; 95% CI 0.56 to 0.77; p < 0.001) or skilled nursing facility (25.3% vs 31.6%; adjusted OR: 0.63; 95% CI 0.47 to 0.84; p = 0.002). There was a trend toward higher hospice utilization in the intervention arm versus control during the 6-month follow-up (12.6% vs 6.8%; adjusted OR: 1.67; 95% CI 0.82 to 3.36; p = 0.15).

**Table 4:** Inpatient and hospice care utilization after discharge (N = 568)

	Control n = 370 % (n)	Intervention n = 198 % (n)	Odds ratio for intervention group (95% CI)^a^	Adjusted p-value^a^
Hospital readmission	45.1% (167)	34.9% (69)	0.66 (0.56–0.77)	< 0.001
LTAC admission	17.0% (63)	13.6% (27)	0.80 (0.49–1.32)	0.38
SNF admission	31.6% (117)	25.3% (50)	0.63 (0.47–0.84)	0.002
Rehabilitation facility admission	31.1% (115)	27.8% (55)	0.87 (0.56–1.37)	0.55
Hospice use	6.8% (25)	12.6% (25)	1.67 (0.82–3.36)	0.15

*LTAC* long-term acute care, *SNF* skilled nursing facility

^a^Results from logistic regression modeling using robust standard error for site-clustering. All models were adjusted for patient’s age, sex, race, modified SAPS III score, Elixhauser index, mechanical ventilation usage, primary diagnosis, and admission source

As previously reported, the cost per patient who received the intervention (N = 547) was $169.88, which included costs associated with the 12-h communication skills training course and regular site visits by a QI implementation specialist throughout the intervention phase [26].

## Discussion

We previously reported that the PARTNER intervention improved surrogates’ ratings of the patient- and family centeredness of care in the ICU, decreased ICU and hospital length of stay, and did not impact surrogates’ psychological symptom burden at 6-month follow-up [26]. In the present analysis, we found that the PARTNER intervention decreased patients’ hospitalization costs, an effect mediated largely by decreased costs among patients who died in the hospital, possibly related to decreased ICU length of stay in that group (4.4 days vs. 6.8 days, as reported previously). In addition, among patients who survived the index hospitalization, the intervention resulted in significantly fewer patients readmitted to acute care hospitals and skilled nursing facilities over 6-month follow-up, with a trend toward increased hospice utilization.

Interventions to support surrogate decision-makers in ICUs have had variable impact on end-of-life treatment intensity and costs. Interventions that have either been brief or focused on providing surrogates better decision-relevant information have generally not impacted index hospitalization costs or healthcare utilization [23, 38, 39]. Conversely, observational data suggests that multifaceted system-level interventions focused on improving serious illness communication may decrease both costs and healthcare use [40, 41]. To the best of our knowledge, only one other family support intervention has demonstrated reduced treatment intensity at the end of life and hospitalization costs in a randomized control trial [42]. That intervention consisted of a trained facilitator who longitudinally supported the family and mediated conflict during the ICU stay. The PARTNER intervention that we tested was also conceptually and practically focused on providing longitudinal emotional and psychological support to surrogates. When taken in context of prior studies, our findings add to the evidence suggesting that interventions focused on providing longitudinal emotional and psychosocial support may be a promising strategy to decrease utilization of invasive burdensome treatments at the end of life. This hypothesis fits with research on surrogates’ experiences that reveals the intense psychological difficulty many experience during the process of making decisions to forego life support for an incapacitate loved one [16, 17, 19].

What may explain that the PARTNER intervention- which was delivered only during the ICU stay- decreased subsequent readmission rates and non-statistically significant trend toward more hospice utilization among survivors through 6-month follow-up? Although our data cannot establish a mechanism, one possibility is that the intervention helped surrogates come to terms with the gravity of their loved one’s illness, such that when the patient experienced a subsequent clinical deterioration, surrogates were more prepared to shift the goals of care to comfort-focused treatment. Another possibility is that the intervention enhanced the readiness of surrogates and patients to engage in advance care planning, which may have resulted in more decisions by patients to forego life support and enter hospice.

In this trial, we included only patients at high risk of death or severe functional impairment, which has implications for how health systems should think about how to apply the trial results to their patient populations. We observed that most of the cost savings of the PARTNER intervention accrued among decedents, but that improvements in the patient- and family-centeredness of care accrued to both survivors and decedents. Therefore, health systems interested in improving patient- and family-centeredness of care might plausibly choose to adopt the intervention among a broader cohort of patients, while acknowledging that the net cost savings would likely be smaller than observed in the trial because doing so would likely require a larger investment in training and increasing nursing staffing.

We also expect that the effect of the PARTNER intervention may be augmented or diminished depending on ICUs’ existing care practices related to family support. For example, the effect of the intervention may be greater in ICUs with less intensive physician staffing models, infrequent interdisciplinary family meetings, and limited integration of specialty palliative care consultants. Conversely, the effect of the intervention may be smaller in ICUs with high intensity intensivist staffing models, protocols for frequent family meetings, and extensive integration of palliative care consultants into patients’ care.

As previously reported, the PARTNER intervention cost approximately $170 USD per patient to deploy, which included the costs of training and ongoing implementation support during the trial [26]. The trial was successfully conducted without increasing nurse staffing in the study ICUs. However, it is possible that ICUs with different nurse staffing models may need to increase staffing to allow nurses the needed time to engage with families. Nonetheless, even under very conservative estimates (e.g., adding 4 h of nursing time for each enrolled patient), the intervention would still result in substantially lower direct variable and total controllable costs.

This study has several strengths. First, the intervention was explicitly designed to be deployed by ICU clinicians, which increases its scalability and lessens the overall costs compared to adding new staff to the ICU team. Second, we leveraged the UPMC health systems activity-based costing system to calculate costs, which arguably provides a more accurate quantification compared to other costing methods. We assessed the impact of the intervention on both costs of the index hospitalization and on utilization of acute inpatient care, skilled nursing and rehabilitation facilities, and hospice after hospital discharge, which provides insight about costs from both the hospital perspective and the payer perspective.

This study also has several limitations. First, our sample was limited to one region of the country. Second, despite randomization, there were baseline differences between groups and, although we used advanced statistical techniques to adjust for these differences, we cannot exclude the possibility of residual confounding. In particular, the intervention group was older and more comorbid, which may have diminished the true effect of the PARTNER intervention on resource utilization due to higher baseline healthcare use associated with increasing age [43]. Third, data on hospitalization cost data was not available on patients admitted before the health system put into place the activity-based costing system due to changes in how UPMC health system recorded cost data. Fourth, we ascertained rates of hospital readmission and utilization of post-acute care facilities through interviews with patients’ family caregivers, which is subject to recall errors.

In conclusion, a low-cost family support intervention delivered by the existing interprofessional ICU team resulted in significant reductions in hospitalization costs, particularly among patients who died. Among patients who survived the index hospitalization, the intervention resulted in fewer patients being subsequently admitted to an acute care hospital or skilled nursing facility and a trend toward more patients enrolling in hospice.

### Supplementary Information

Supplementary Material 1.

## Data Availability

The datasets used and/or analyzed during the current study are available from the corresponding author on reasonable request.

## Abbreviations

ICU: Intensive Care Medicine
PARTNER: Pairing Re-engineered ICU Teams with Nurse-Driven Emotional Support and Relationship-Building
SAPS: Simplified Acute Physiology Score
UPMC: University of Pittsburgh Medical Centre
