# Supplementary material for: Impact of a family support intervention on hospitalization costs and hospital readmissions among ICU patients at high risk of death or severe functional impairment
Source: Ann Intensive Care. 2024 Jul 2;14:103. doi: 10.1186/s13613-024-01344-9 (PMC11219699; doi:10.1186/s13613-024-01344-9)
Supplement: Supplementary file 1 — Supplementary Material 1. [file 13613_2024_1344_MOESM1_ESM.docx]

**Supplemental Table 1.** **Characteristics of enrolled patients and surrogates with cost data (N=1012)**

| **Patient Characteristic** | **Control**  **(n=484)** | **Intervention**  **(n=528)** | **p-value^a^** | **Total**  **(N=1012)** |
| --- | --- | --- | --- | --- |
| Age, mean (SD) | 62.5 (15.4) | 67.1 (14.9) | <0.001 | 64.9 (15.3) |
| Female, count (%) | 233 (48.1) | 282 (53.4) | 0.094 | 515 (50.9) |
| Race, count (%)  White  Black  Hispanic  Other  Not documented | 383 (79.1)  41 (8.5)  2 (0.4)  4 (0.8)  54 (11.2) | 444 (84.1)  40 (7.6)  0 (0.0)  4 (0.8)  40 (7.6) | 0.153 | 827 (81.7)  81 (8.0)  2 (0.2)  8 (0.8)  94 (9.3) |
| Primary Diagnosis, count (%)  Other  Cardiovascular  Pulmonary  GI  Toxicology  Infection/Sepsis  Neurological  Oncological | 65 (13.5)  13 (2.7)  63 (13.1)  60 (12.5)  22 (4.6)  113 (23.5)  103 (21.4)  42 (8.7) | 55 (10.4)  33 (6.3)  100 (19.0)  47 (8.9)  17 (3.2)  152 (28.8)  105 (19.9)  18 (3.4) | <0.001 | 120 (11.9)  46 (4.6)  163 (16.2)  107 (10.6)  39 (3.9)  265 (26.3)  208 (20.6)  60 (6.0) |
| Admission source, count (%)  Direct  Emergency  Other hospital  Skilled nursing facility | 131 (27.1)  287 (59.3)  66 (13.6)  0 (0.0) | 49 (9.3)  404 (76.5)  73 (13.8)  2 (0.4) | <0.001 | 180 (17.8)  691 (68.3)  139 (13.7)  2 (0.2) |
| Modified SAPS III score, mean (SD) | 50.5 (12.3) | 50.9 (11.8) | 0.605 | 50.7 (12.1) |
| Elixhauser comorbidity index (count; range 0-29), mean (SD) | 5.1 (2.3) | 5.8 (2.4) | <0.001 | 5.4 (2.4) |
| On mechanical ventilation during hospitalization, count (%) | 432 (89.3) | 461 (87.3) | 0.337 | 893 (88.2) |
| **Surrogate Characteristic** | **Control**  **(n=363)** | **Intervention**  **(n=411)** | **p-value^a^** | **Total**  **(N=774)^b^** |
| Age, mean (SD) | 55.4 (13.5) | 57.0 (13.7) | 0.102 | 56.2 (13.7) |
| Female, count (%) | 266 (74.1) | 273 (66.9) | 0.030 | 539 (70.3) |
| Race, count (%)  White  Black  Hispanic  Asian  Multiethnic | 309 (90.9)  22 (6.5)  4 (1.2)  4 (1.2)  1 (0.3) | 367 (90.4)  35 (8.6)  0 (0.0)  2 (0.5)  2 (0.5) | 0.128 | 676 (90.6)  57 (7.6)  4 (0.5)  6 (0.8)  3 (0.4) |
| Relationship to Patient, count (%)  Spouse/partner  Parent  Child  Sibling  Other relative  Other relationship | 145 (39.9)  41 (11.3)  114 (31.4)  38 (10.5)  13 (3.6)  12 (3.3) | 155 (37.9)  28 (6.9)  158 (38.6)  50 (12.2)  9 (2.2)  9 (2.2) | 0.076 | 300 (38.9)  69 (8.9)  272 (35.2)  88 (11.4)  22 (2.9)  21 (2.7) |

^a^From Student’s t-test or Pearson's chi-squared test

^b^Total number of surrogates who responded to at least one demographic questions

**Supplemental Table 2.** **Characteristics of enrolled patients and surrogates with follow-up data (N=809)**

| **Patient Characteristic** | **Control**  **(n=501)** | **Intervention**  **(n=308)** | **p-value^a^** | **Total**  **(N=809)** |
| --- | --- | --- | --- | --- |
| Age, mean (SD) | 63.2 (15.7) | 67.6 (15.1) | <0.001 | 64.9 (15.6) |
| Female, count (%) | 222 (44.3) | 164 (53.3) | 0.013 | 386 (47.7) |
| Race, count (%)  White  Black  Hispanic  Other  Not documented | 414 (82.6)  25 (5.0)  1 (0.2)  4 (0.8)  57 (11.4) | 260 (84.4)  23 (7.5)  0 (0.0)  1 (0.3)  24 (7.8) | 0.214 | 674 (83.3)  48 (5.9)  1 (0.1)  5 (0.6)  81 (10.0) |
| Primary Diagnosis, count (%)  Other  Cardiovascular  Pulmonary  GI  Toxicology  Infection/Sepsis  Neurological  Oncological | 75 (15.0)  17 (3.4)  82 (16.4)  53 (10.6)  22 (4.4)  116 (23.3)  101 (20.2)  33 (6.6) | 33 (10.8)  24 (7.8)  61 (19.9)  29 (9.5)  7 (2.3)  81 (26.4)  63 (20.5)  9 (2.9) | 0.007 | 108 (13.4)  41 (5.1)  143 (17.7)  82 (10.2)  29 (3.6)  197 (24.4)  164 (20.4)  42 (5.2) |
| Admission source, count (%)  Direct  Emergency  Other hospital  Skilled nursing facility | 137 (27.4)  303 (60.5)  61 (12.2)  0 (0.0) | 28 (9.1)  246 (79.9)  32 (10.4)  2 (0.7) | <0.001 | 165 (20.4)  549 (67.9)  93 (11.5)  2 (0.3) |
| Modified SAPS III score, mean (SD) | 49.5 (11.9) | 50.3 (11.5) | 0.359 | 49.8 (11.8) |
| Elixhauser comorbidity index (count; range 0-29), mean (SD) | 5.0 (2.5) | 5.7 (2.4) | <0.001 | 5.3 (2.5) |
| On mechanical ventilation during hospitalization, count (%) | 439 (87.6) | 273 (88.6) | 0.667 | 712 (88.0) |
| **Surrogate Characteristic** | **Control**  **(n=499)** | **Intervention**  **(n=307)** | **p-value^a^** | **Total**  **(N=806)^b^** |
| Age, mean (SD) | 57.5 (13.1) | 57.8 (13.2) | 0.727 | 57.6 (13.2) |
| Female, count (%) | 375 (75.8) | 207 (67.7) | 0.012 | 582 (72.7) |
| Race, count (%)  White  Black  Hispanic  Asian  Multiethnic | 425 (92.2)  24 (5.2)  5 (1.1)  5 (1.1)  2 (0.4) | 279 (91.5)  24 (7.9)  0 (0.0)  1 (0.3)  1 (0.3) | 0.147 | 704 (91.9)  48 (6.3)  5 (0.7)  6 (0.8)  3 (0.4) |
| Relationship to Patient  Spouse/partner  Parent  Child  Sibling  Other relative  Other relationship | 219 (43.9)  47 (9.4)  134 (26.9)  68 (13.6)  14 (2.8)  17 (3.4) | 117 (38.1)  22 (7.2)  114 (37.1)  39 (12.7)  8 (2.6)  7 (2.3) | 0.071 | 336 (41.7)  69 (8.6)  248 (30.8)  107 (13.3)  22 (2.7)  24 (3.0) |

^a^From Student’s t-test or Pearson's chi-squared test

^b^Total number of surrogates who responded to at least one demographic questions
